# Evaluating the Effectiveness and Safety of Vedolizumab in Moderate to Severe Ulcerative Colitis: A Systematic Review and Meta-Analysis of Randomized Controlled Trials

**DOI:** 10.7759/cureus.90352

**Published:** 2025-08-17

**Authors:** Abdulraouf Flemban, Hamzah A Bakhor, Raghad Almuzaini, Taif Alshahrani, Raghid A Alahmadi, Raneem Alshurbi, Khaldah Althobiti, Hams Alwadai, Sufyan Maghrabi, Najwa Alkhairy, Moath Shawosh, Taghreed Zamzami

**Affiliations:** 1 Pharmacy, Umm Al-Qura University, Makkah, SAU; 2 Medicine, Ibn Sina National College, Jeddah, SAU; 3 Pharmacy, Taibah University, Madinah, SAU; 4 Medicine, King Khalid University, Abha, SAU; 5 Medicine, Taif University, Taif, SAU; 6 Medicine, King Faisal Medical Complex, Taif, SAU; 7 Medicine, Imam Mohammad Ibn Saud Islamic University, Riyadh, SAU; 8 Medicine, King Abdulaziz University, Jeddah, SAU; 9 Medicine, Umm Al-Qura University, Makkah, SAU; 10 Medicine and Surgery, Faculty of Medicine, University of Jeddah, Jeddah, SAU; 11 Clinical Pharmacy, King Abdullah Medical City, Makkah, SAU

**Keywords:** clinical remission, mucosal healing, safety, ulcerative colitis, vedolizumab

## Abstract

Ulcerative colitis (UC) significantly affects patients' lives, and treatment options may be constrained due to severe side effects. Vedolizumab, a monoclonal antibody that specifically targets the α4β7 integrin, has recently demonstrated potential as a gut-specific therapy with fewer adverse effects compared to other biological treatments. We conducted a systematic review and meta-analysis to evaluate the efficacy and safety of vedolizumab in adults with moderate to severe UC. The research team reviewed three prominent databases - PubMed, Google Scholar, and Cochrane Central Register of Controlled Trials (CENTRAL) - adhering to Preferred Reporting Items for Systematic reviews and Meta-Analyses (PRISMA) criteria, to identify randomized controlled trials that compared vedolizumab with placebo or other treatments for moderate to severe UC. A total of 1,571 preliminary studies were screened, resulting in the inclusion of six trials with 1,618 people. The primary outcomes included clinical remission, mucosal healing, clinical response, corticosteroid-free remission, and adverse events such as infections and infusion site responses. Data indicate that vedolizumab markedly enhanced endoscopic remission relative to placebo, nearly doubling the likelihood of remission for patients (risk ratio (RR) = 2.25; p = 0.01). Despite the prevalence of clinical remission in the vedolizumab cohort, this outcome lacks statistical significance (RR = 2.10; p = 0.07). No substantial advantage was observed in corticosteroid-free remission (RR = 1.69; p = 0.58). Vedolizumab exhibited an acceptable safety profile, with adverse event rates comparable to those of a placebo. Nonetheless, minor infections and infusion responses occurred with slightly greater frequency. Vedolizumab enhances endoscopic healing and clinical response in individuals with moderate to severe UC while maintaining an acceptable safety profile. This therapeutic option is particularly significant for individuals who experience difficulties with alternative therapies. Future head-to-head trials comparing vedolizumab with other biological therapies may elucidate its role in treatment regimens.

## Introduction and background

Ulcerative colitis (UC) is a chronic inflammatory bowel disease marked by inflammation of the mucosal and submucosal layers of the colon, resulting in rectal inflammation and ulceration [1]. The primary etiology of UC is idiopathic; however, various risk factors have been identified that contribute to its pathogenesis, including genetic predisposition, immune system dysregulation, environmental influences, and changes in gut microbiota [2-4]. In specific instances, extra-intestinal manifestations, including arthritis, may arise [5]. Severe instances of UC may result in complications such as colorectal cancer [6].

Diverse therapeutic classes are utilized in the treatment of UC, including aminosalicylates (5-ASA), corticosteroids, biologic therapies such as infliximab and vedolizumab, and non-biologic immunomodulators like azathioprine [7-9]. For patients with severe UC who do not respond to these treatments, surgery is the final option. Surgical interventions, however, entail risks of post-operative complications including infections, anastomotic leakage, and pelvic sepsis [10]. This highlights the persistent necessity for investigation into innovative therapeutic alternatives that can effectively manage moderate to severe UC while reducing treatment-related adverse effects.

Vedolizumab is a humanized monoclonal IgG1 antibody that specifically binds to the α4β7 integrin present on lymphocyte surfaces [11]. Vedolizumab disrupts the inflammatory cascade in the gut by inhibiting lymphocyte migration to the gut-associated lymphoid tissue (GALT) and the intestinal lamina propria. This mechanism provides a gut-selective therapeutic strategy linked to reduced systemic side effects relative to alternative biologic treatments [12,13].

This systematic review and meta-analysis seek to assess the safety and efficacy of vedolizumab for the treatment of moderate to severe UC in adult patients. This study evaluates safety parameters, including side effects and infection rates, as well as key clinical outcomes such as remission rates, mucosal healing, clinical response, and corticosteroid-free remission.

## Review

Materials and methods

This systematic review and meta-analysis adhered to the Preferred Reporting Items for Systematic Reviews and Meta-Analyses (PRISMA) guidelines.

Search Strategy

We conducted a systematic search of PubMed, Google Scholar, and the Cochrane Central Register of Controlled Trials (CENTRAL). The search employed keywords and medical subject headings related to UC, colitis, vedolizumab, α4β7 integrin inhibitor, Entyvio, and randomized controlled trials (RCTs).

Eligibility Criteria

We incorporated randomized, placebo-controlled trials that assessed the efficacy and safety of vedolizumab in individuals with moderate to severe UC.

The PICO Criteria

The PICO criteria for this review were defined as follows: P (Population): adults diagnosed with moderate to severe UC; I (Intervention): administration of vedolizumab, irrespective of dosage or formulation; C (Comparator): placebo, alternative biologic therapies (such as anti-TNF agents), or standard treatments; and O (Outcomes): clinical remission, mucosal healing, clinical response, corticosteroid-free remission, and safety outcomes (adverse events and infections).

Exclusion Criteria

Studies were excluded based on the following conditions: inclusion of patients with mild UC or pediatric populations, presence of comorbid health conditions unrelated to UC, failure to utilize vedolizumab as the primary intervention, absence of a valid comparator group for analysis, and lack of data addressing primary safety or efficacy outcomes.

Study designs that were non-randomized, including observational studies, reviews, case reports, or preclinical trials, were also excluded.

Outcome Measures

The primary efficacy outcomes were clinical remission, mucosal healing, clinical response, and remission without corticosteroids. The safety outcomes encompassed the incidence of infections and other adverse events that occurred.

Selection of Studies, Extraction of Data, and Evaluation of Quality

Rayyan software (Rayyan Systems Inc., Cambridge, MA) was utilized for screening and de-duplication purposes. Two independent reviewers screened the titles and abstracts, with any discrepancies resolved by a third reviewer. Two reviewers evaluated the records, with disputes adjudicated by a third reviewer.

Disputes are settled through dialogue. The documentation included the screening, randomization, follow-up periods, and the inclusion and exclusion criteria.

The demographics of participants encompassed age and gender. The outcome metrics, total participant count, group allocations, analytical cohorts, and safety parameters were recorded.

**Figure 1:**
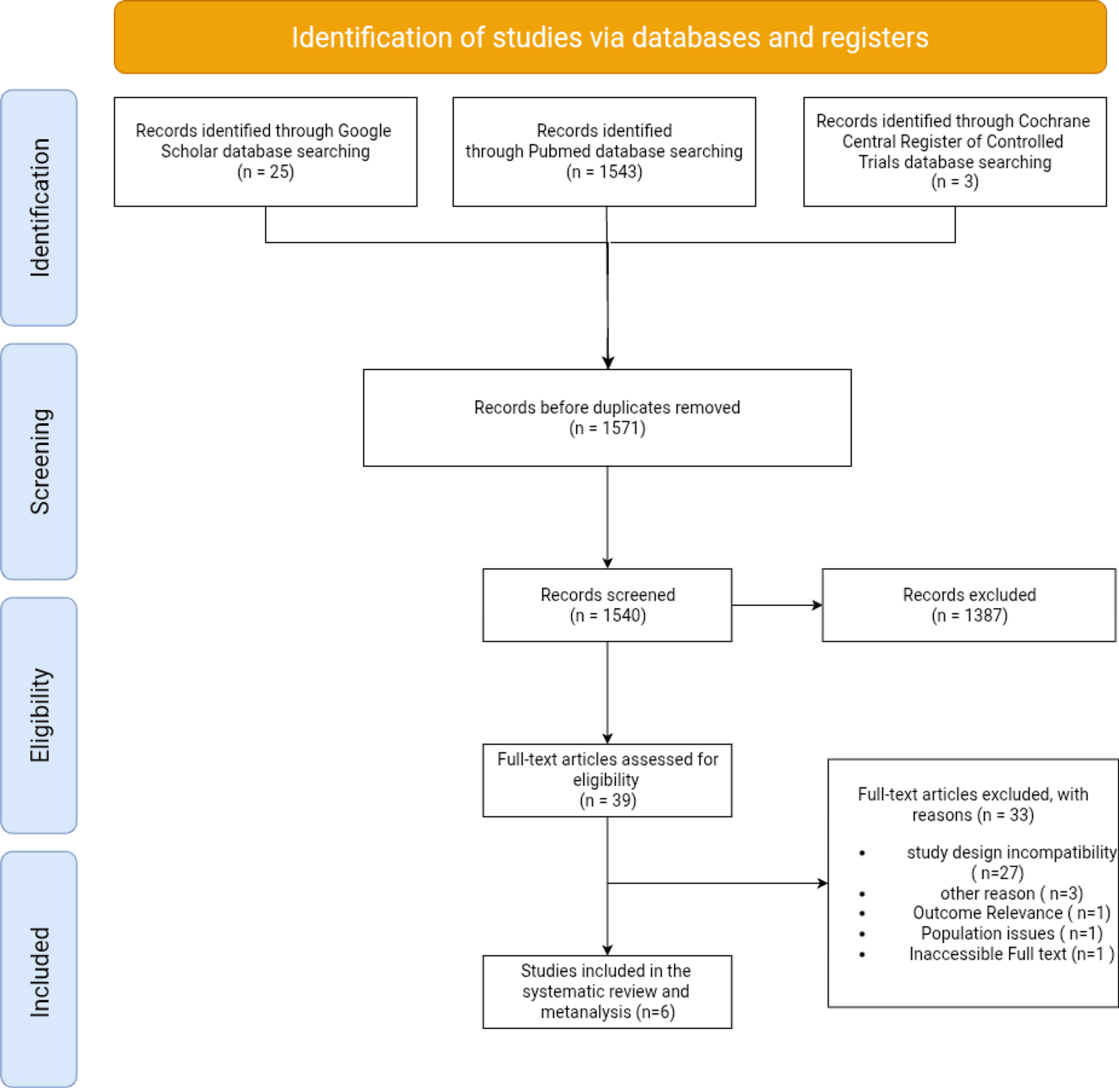
Flow diagram for the systematic review.

Two authors independently evaluated the risk of bias utilizing the Cochrane Risk of Bias 2 (RoB 2) tool within Review Manager 5.4 software (The Cochrane Collaboration, Oxford, UK), and any disagreements were addressed through discussion or consultation with a third author [14]. The certainty of evidence was assessed utilizing GRADE. The evidence was diminished due to significant or grave concerns [15]. PRISMA flow diagram for the systematic review (Figure 1).

Statistical Analysis

The heterogeneity of the included studies was evaluated using funnel plots, chi-squared tests, and I² statistics. If there was no substantial heterogeneity (p > 0.05), a fixed-effect model was applied to synthesize the results [16,17]. Conversely, if the p-value was 0.05 or less, the choice between a fixed-effect or random-effects model was determined based on the I² value: a fixed-effect model was used when I² ≤ 40%, and a random-effects model was employed when I² ≥ 40% [18-21].

The mean difference (MD) and risk ratio (RR) were utilized to assess the relationship between the intervention and continuous or dichotomous outcomes, with corresponding 95% confidence intervals (CIs) provided. All efficacy analyses were conducted following a modified intention-to-treat approach, including all participants who were randomly assigned and received at least one dose of the study intervention.

Safety and tolerability analyses included all participants who received at least one dose of the study drug. All reported p-values were two-sided, with statistical significance defined as p < 0.05. Data analysis was performed using the Review Manager 5.4 software.

Results

Study Selection and Characteristics

The systematic review and meta-analysis initially identified 1,571 citations through database searches. These comprised 87 from PubMed, three from CENTRAL, and 25 from Google Scholar. After eliminating duplicates, 1,540 studies remained for title and abstract screening. Subsequent to the initial screening, 1,387 studies were excluded, leaving only 39 full-text articles for examination. Following a comprehensive evaluation, only six studies satisfied the inclusion criteria and were incorporated into the final meta-analysis.

Patient Demographics and Baseline Characteristics

A total of 1,618 patients with moderate to severe UC were included in the six RCTs. Each study included anywhere from 181 to 895 patients. Participants in the studies ranged in age from 18 to 80, and the average age varied from study to study. In the study by Lattanzi et al. [19], the average age was between 38.1 and 40.5 years, while in Ashraf et al. [22], it was between 42.8 and 43.4 years. An equal number of men and women participated in each trial. According to Lattanzi et al. [19], there were 65 men and 41 women in the vedolizumab SC group and 33 men and 23 women in the placebo group. The average length of the illness varied from six to 10 years, depending on the study: 8.0 years (subcutaneous (SC)), 8.4 years (intravenous (IV)), and 7.9 years (placebo) were reported by Lattanzi et al. [19].

To distinguish between moderate and severe UC, all studies used endoscopic and clinical Mayo scores. Many of the patients had previously received treatment with biologics, immunomodulators, or corticosteroids. According to Ashraf et al. [22], 34-36% of patients were "anti-TNF naïve." There were conflicting reports about smoking status; Lattanzi et al. [19] found that 10.4% of patients on vedolizumab SC were smokers. Across all studies, the average body weight was between 74.8 and 76.4 kg.

Intervention Details

The six studies assessed vedolizumab in both IV and SC formulations, comparing it to either placebo or active comparators. Dosing protocols differed among studies. The predominant regimen utilized consisted of IV vedolizumab administered at a dosage of 300 mg every eight weeks. Conversely, subcutaneously administered vedolizumab was given at a dosage of 108 mg biweekly, as assessed in the study by Lattanzi et al. [19]. Additionally, Ashraf et al. [22] investigated dose optimization by contrasting IV vedolizumab 300 mg given every four weeks with administration every eight weeks.

Administration schedules typically conformed to a standard IV induction protocol, with doses given at weeks 0, 2, and 6, followed by maintenance dosing every four or eight weeks. In contrast, SC vedolizumab was administered regularly on a biweekly schedule. Treatment durations varied from 10 weeks during induction phases to a maximum of 52 weeks in maintenance phases.

Control groups comprised both placebo and active comparators. Lattanzi et al. [19] specifically compared SC vedolizumab to IV vedolizumab, whereas Ashraf et al. [22] evaluated standard IV dosing against optimized dosing regimens. Most trials allowed the use of concomitant medications, such as mesalamine, corticosteroids, and immunomodulators.

Despite inconsistent reporting of treatment adherence, retention rates during maintenance phases were predominantly elevated. Vedolizumab was administered according to standardized protocols across the studies included, ensuring comparability within this systematic review and meta-analysis.

Clinical Outcomes

**Figure 2:**
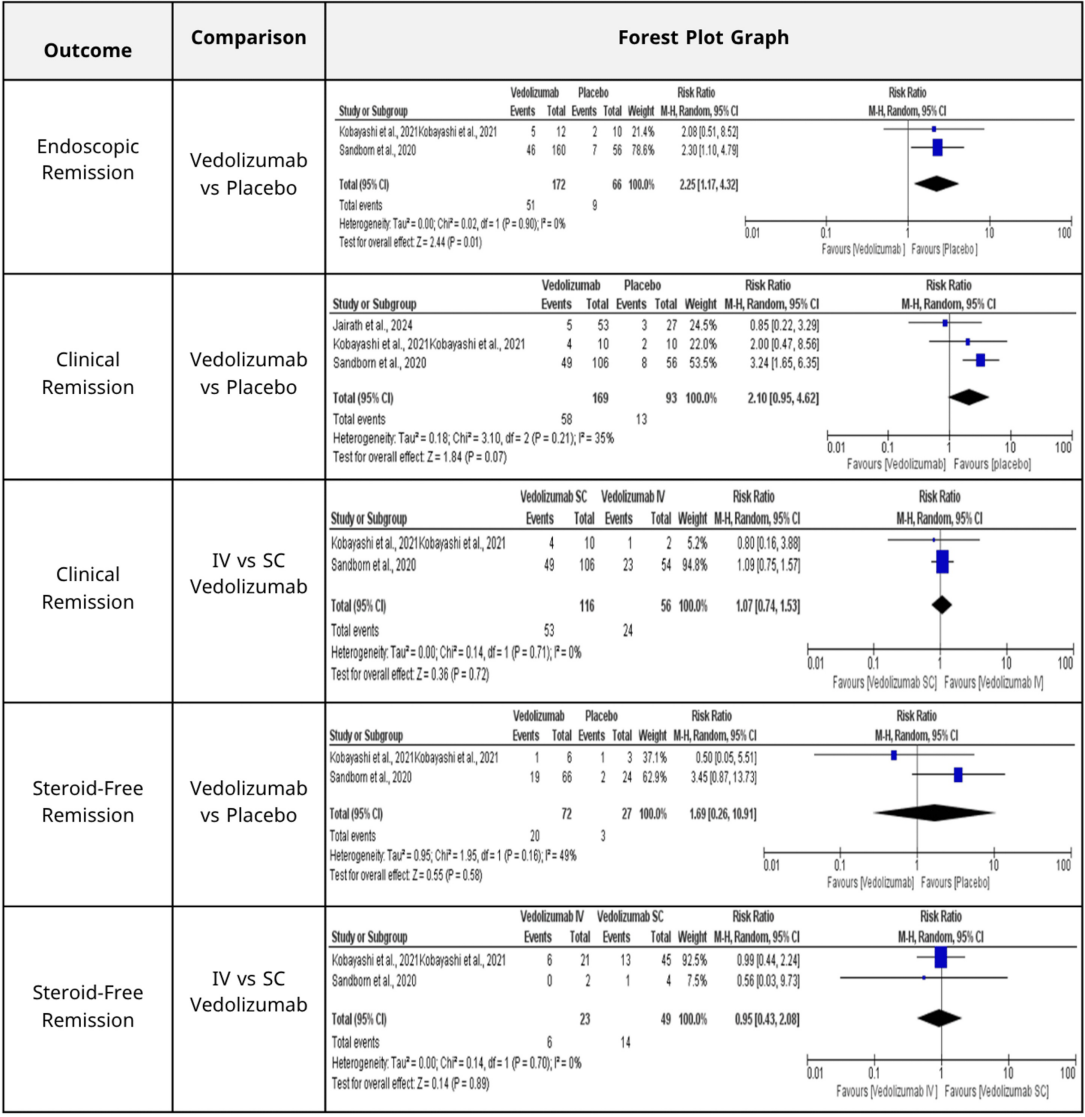
Summary of the pooled risk ratios and heterogeneity measures for clinical and endoscopic outcomes, alongside corresponding forest plots.

Primary efficacy outcomes: The analysis included two studies [15,19] that assessed vedolizumab compared to placebo for attaining endoscopic remission. The results indicated a statistically significant enhancement associated with vedolizumab, yielding an RR of 2.25 (95% CI: 1.17-4.32; p = 0.01). The absence of heterogeneity (I² = 0%) signifies a high degree of consistency among the included studies (Figure 2).

Three studies [15,19,22] evaluated vedolizumab against placebo for clinical remission. Despite vedolizumab demonstrating a tendency for enhancement (RR = 2.10, 95% CI: 0.95-4.62; p = 0.07), the outcome did not attain statistical significance. The variability among these studies was moderate (I² = 35%) (Figure 2).

Two studies [15,19] were analyzed for the comparison of IV and SC administration of vedolizumab. The findings indicated no substantial difference between the two administration routes (RR = 1.07, 95% CI: 0.74-1.53; p = 0.72), with no detected heterogeneity (I² = 0%) (Figure 2).

Concerning corticosteroid-free remission, two studies [15,19] evaluated vedolizumab against placebo. The findings were not statistically significant (RR = 1.69, 95% CI: 0.26-10.91; p = 0.58), exhibiting moderate heterogeneity (I² = 49%) (Figure 2). In a comparable analysis of vedolizumab IV versus SC administration, the pooled results [15,19] demonstrated no statistically significant difference (RR = 0.95, 95% CI: 0.43-2.08; p = 0.89; I² = 0%) (Figure 2).

Similarly, the comparison between vedolizumab IV and SC revealed consistently insignificant differences in the pooled results (RR = 0.95, 95% CI: 0.43-2.08; p = 0.89; I² = 0%) (Figure 2).

**Figure 3:**
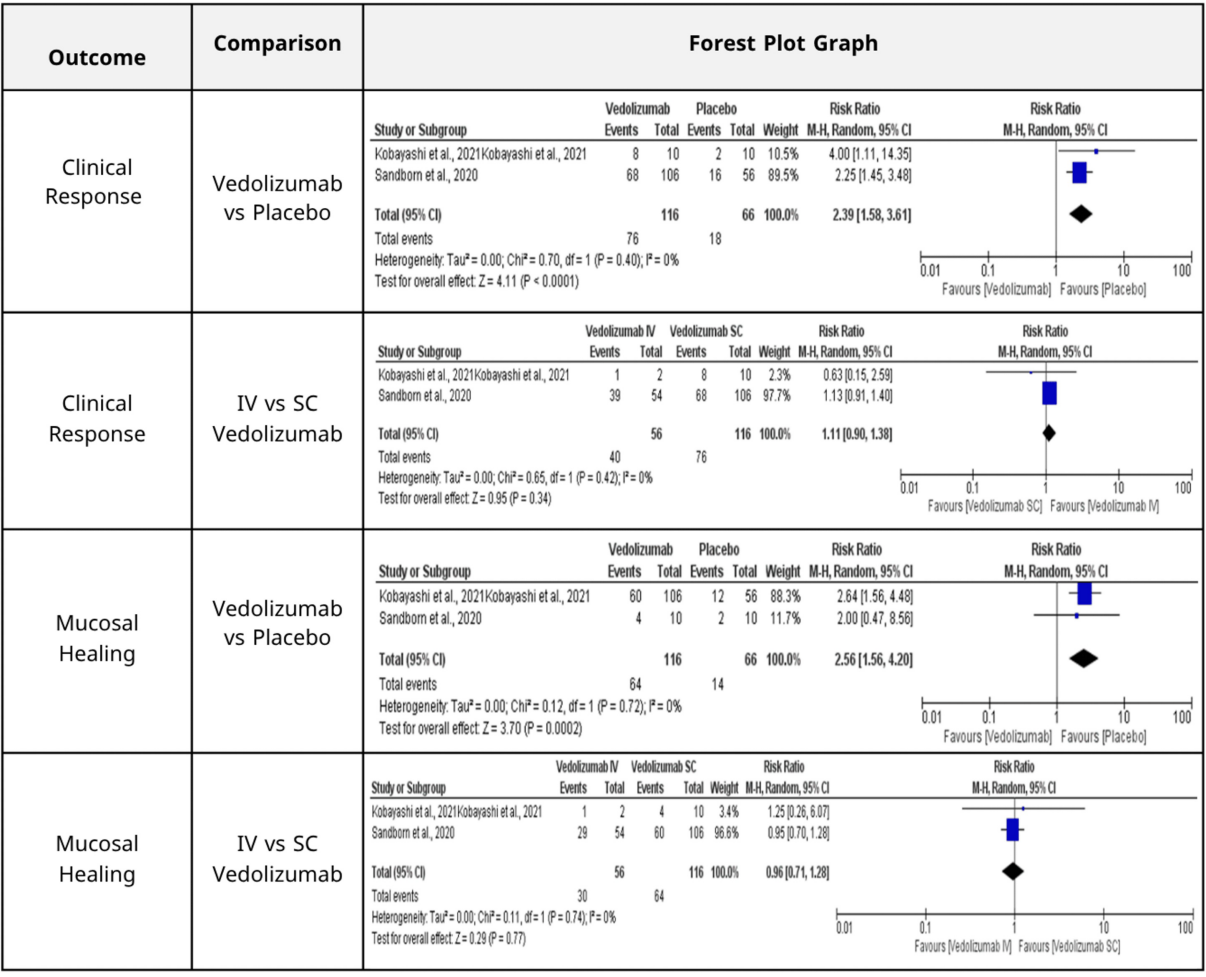
Summary of the comparative effects of vedolizumab versus placebo and intravenous (IV) versus subcutaneous (SC) administration on clinical response and mucosal healing outcomes. Statistically significant differences were observed in favor of vedolizumab over placebo for both outcomes, while no significant differences were noted between the administration routes.

Secondary efficacy outcomes: Vedolizumab markedly enhanced the clinical response relative to placebo, as depicted in (Figure 3) (RR = 2.39, 95% CI: 1.58-3.61; p < 0.0001; I² = 0%), indicating substantial consistency across studies [15,19]. No significant difference was noted between SC and IV administration routes of vedolizumab (Figure 3) (RR = 1.11, 95% CI: 0.90-1.38; p = 0.34; I² = 0%).

Vedolizumab exhibited a statistically significant enhancement in mucosal healing relative to placebo (Figure 3) (RR = 2.56, 95% CI: 1.56-4.20; p = 0.0002; I² = 0%). The comparison of IV and SC formulations demonstrated no statistically significant difference (Figure 3) (RR = 0.96, 95% CI: 0.71-1.28; p = 0.77; I² = 0%) [15,19].

In addition to clinical and endoscopic outcomes, vedolizumab was linked to decreased hospitalization rates relative to placebo [21]. Moreover, fecal calprotectin concentrations markedly diminished in patients administered vedolizumab [15,19,21,22].

**Figure 4:**
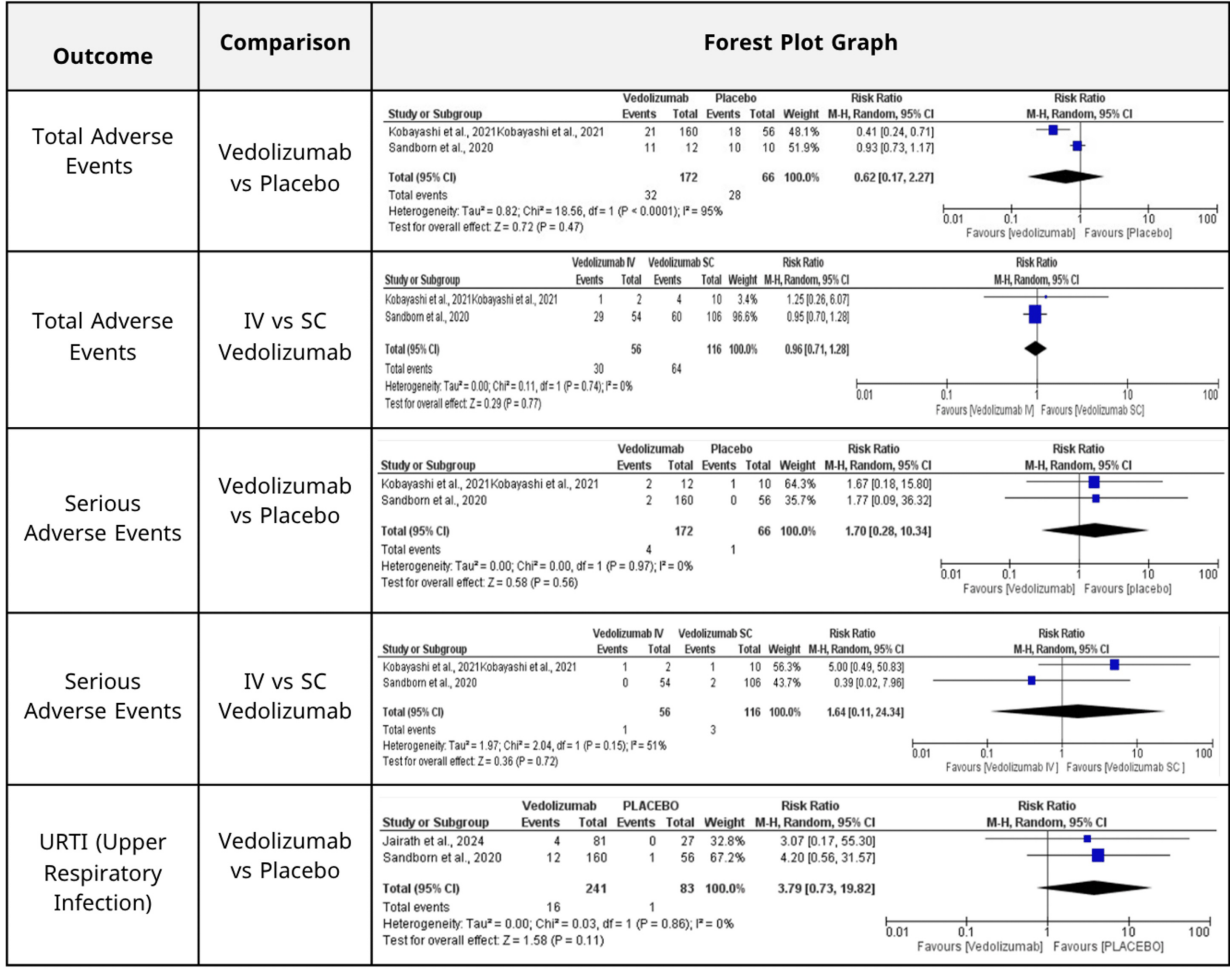
Safety outcome comparisons, including total and serious adverse events, and the incidence of upper respiratory tract infection. No statistically significant differences were observed between vedolizumab and placebo, nor between intravenous (IV) and subcutaneous (SC) administration routes across all outcomes.

Safety outcomes: Vedolizumab did not exhibit a statistically significant increase in the incidence of overall adverse events compared to placebo, as depicted in (Figure 4) (RR = 0.62, 95% CI: 0.17-2.27; p = 0.47; I² = 95%). A considerable degree of heterogeneity was observed among the included studies [15,19]. No significant difference in safety outcomes was detected between IV and SC formulations of vedolizumab (Figure 4) (RR = 0.91, 95% CI: 0.57-1.46; p = 0.70; I² = 0%).

Regarding serious adverse events, vedolizumab did not exhibit a statistically significant increase compared to placebo (Figure 4) (RR = 1.70, 95% CI: 0.28-10.34; p = 0.56; I² = 0%). Likewise, no substantial difference was observed when comparing IV and SC formulations (Figure 4) (RR = 1.64, 95% CI: 0.11-24.34; p = 0.72; I² = 51%) (15,19).

Upper respiratory tract infections (URTIs) were more prevalent in patients administered vedolizumab compared to those receiving a placebo (Figure 4) (RR = 3.79, 95% CI: 0.73-19.82; p = 0.11; I² = 0%). This finding, however, was accompanied by a wide confidence interval, indicating variability [19,22].

Infusion-related reactions were primarily mild, with recorded injection-site reactions such as rash, swelling, and erythema in specific studies [19]. In contrast, alternative studies did not report notable adverse events associated with infusion [15,21,22].

Risk of Bias and Study Quality Assessment

**Figure 5:**
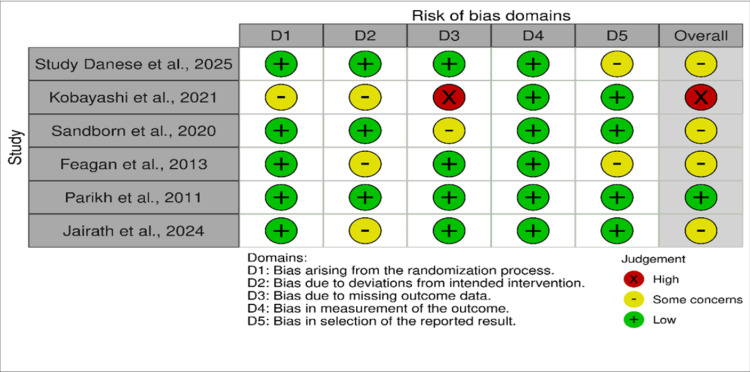
Bias assessment of the included studies.

Two independent evaluators assessed the risk of bias utilizing the Cochrane Risk of Bias tool (RoB 2), examining five critical domains within the included RCTs. The evaluation determined that one study [15] exhibited a significant risk of bias, chiefly owing to issues concerning randomization and the management of absent data. Conversely, the other studies demonstrated a low to moderate risk of bias, thus ensuring overall methodological reliability. The overall risk of bias assessment across the included studies is presented in Figure 5.

Discussion

UC is a type of inflammatory bowel disease, primarily idiopathic, marked by inflammation of the mucosa and submucosa of the colon, which may result in symptoms including abdominal pain, tenesmus, mucus discharge, and hematochezia. Treatment for UC encompasses 5-ASA, corticosteroids, biologics like infliximab and vedolizumab, non-biologics like azathioprine, and surgical intervention in severe cases [23]. One treatment option is a novel class of biological drugs known as anti-adhesion molecule inhibitors, exemplified by vedolizumab, a humanized monoclonal IgG1 antibody that specifically targets the α4β7 integrin on lymphocyte surfaces.

The evaluation of vedolizumab compared to placebo for endoscopic remission demonstrated a statistically significant enhancement with vedolizumab. Likewise, both clinical response and mucosal healing exhibited substantial enhancement. There was no statistical significance between clinical remission and steroid-free remission. Vedolizumab demonstrated no notable elevation in total or severe adverse events relative to placebo. Nonetheless, URTIs occurred more frequently in patients treated with vedolizumab compared to those receiving placebo, and infusion-related reactions were predominantly mild. The findings of a systematic review concerning the safety and efficacy of biological therapies for inflammatory bowel disease corroborate the results pertaining to vedolizumab [22], thereby reinforcing our conclusions.

A limitation is the exclusive reliance on freely accessible English-language sources, potentially resulting in the oversight of significant studies published in other languages or behind paywalls. Furthermore, we excluded pediatric populations; thus, our findings pertain solely to adults, creating a deficiency in comprehending vedolizumab’s safety and efficacy in younger individuals. Moreover, the absence of direct comparisons with alternative biological therapies complicates the identification of the optimal treatment strategy. Notwithstanding these constraints, our results provide significant insights into vedolizumab's function in the management of moderate to severe UC. Subsequent research ought to concentrate on direct comparisons with alternative treatments to enhance comprehension of their relative efficacy. Cost-effectiveness analyses are necessary to evaluate its integration within healthcare budgets. In addition to clinical trials, real-world studies and post-marketing surveillance can offer a more comprehensive understanding of the drug's efficacy among varied patient populations. These will enhance treatment strategies and facilitate evidence-based decision-making for patients and healthcare providers.

## Conclusions

We conducted a systematic review and meta-analysis to evaluate the safety and efficacy of vedolizumab in individuals with moderate to severe UC. Our research indicates that vedolizumab enhances mucosal healing, elevates clinical response, promotes endoscopic remission, and reduces hospitalization rates.

Nonetheless, certain adverse effects are associated with the treatment, including injection site reactions, infusion-related responses, and URTIs. We recommend that future research should focus on direct comparisons with other biologic treatments to more clearly delineate vedolizumab's relative efficacy.
